# Hypofractionated Gamma Knife Icon Radiosurgery for Perioptic Meningiomas: Clinical and Radiological Outcomes in a Series of 100 Patients

**DOI:** 10.3390/life16050781

**Published:** 2026-05-07

**Authors:** Karol Migliorati, Lodoviga Giudice, Clarissa Ferrari, Chiara Zani, Giorgio Spatola, Chiara Bassetti, Nicola Redolfi, Corrado D’Arrigo, Rosaria Maio, Matteo Chieregato, Cesare Giorgi, Mario Bignardi, Alberto Franzin

**Affiliations:** 1 Gamma Knife Unit, Department of Neurosurgery, Fondazione Poliambulanza Istituto Ospedaliero, 25124 Brescia, Italy; karol.migliorati@poliambulanza.it (K.M.);; 2 Research and Clinical Trials Office, Fondazione Poliambulanza Istituto Ospedaliero, 25124 Brescia, Italy; 3 Department of Neurosurgery, Fondazione IRCCS San Gerardo dei Tintori, 20900 Monza, Italy; 4 Unit of Medical Physics, ASST Cremona, 26100 Cremona, Italy; 5 Unit of Medical Physics, Fondazione Poliambulanza Istituto Ospedaliero, 25124 Brescia, Italy; 6 Department of Radiation Oncology, Fondazione Poliambulanza Istituto Ospedaliero, 25124 Brescia, Italy

**Keywords:** hypofractionated radiosurgery, Gamma Knife Icon, perioptic meningiomas

## Abstract

Perioptic meningiomas pose a therapeutic challenge due to their proximity to critical visual structures. Single-fraction stereotactic radiosurgery is known to effectively control the growth of meningiomas, but this subgroup carries the risk of optic neuropathy, which is minimized with the introduction of dose hypofractionation. The Leksell Gamma Knife Icon has perfected fractionated stereotactic radiosurgery, maintaining submillimeter accuracy in each dose fraction without the need for an invasive frame. This study analyzes the feasibility, safety, and efficacy of multi-fraction Gamma Knife Icon radiosurgery for perioptic meningiomas, taking into account tumor control rates, visual preservation, and treatment-related toxicity. We conducted a retrospective analysis of 100 patients with a perioptic meningioma treated with fractionated Gamma Knife Icon radiosurgery between September 2017 and December 2022. A total of 80 Patients were female, and 20 were male; the mean age was 61.7 years (range 35–84). The most frequent anatomical locations included: cavernous sinus (35 pts), anterior clinoid (17 pts), sphenoid wing (14 pts) and olfactory groove (11 pts). The median tumor volume was 5.6 mL (range 0.12–31.7 mL). Most patients (89%) received 25 Gy in five fractions. Tumor control was achieved in 98% of cases, with a mean radiological follow-up of 41.2 months. Tumor volume did not predict radiological shrinkage (*p* = 0.639). Tumor shrinkage was observed more frequently in the no prior surgery group (*p* = 0.035). The mean clinical follow-up was 45.3 months. Among symptomatic patients (35 pts) at baseline, symptoms remained stable in 27 (77%) cases, improved in 5 (14%), and worsened in 3 (9%). No new symptoms were observed in asymptomatic patients. Overall clinical deterioration occurred in three (3%) patients—one because of tumor progression; although, without statistical evidence (*p* = 0.217), worsened patients had notable larger mean tumor volumes (12.6 mL vs. 6.8 mL). The dosimetric advantages of Gamma Knife technology are empowered by the biological benefits of fractionation and the convenience of non-invasive immobilization. Excellent tumor control rates and positive visual outcomes favor its routine application in properly selected patients.

## 1. Introduction

Perioptic meningiomas represent a distinct therapeutic challenge, constituting approximately 10% of all intracranial meningiomas [1]. These neoplasms arise in close proximity to the optic nerves, optic chiasm, and adjacent neurovascular structures, making surgical resection technically demanding and often associated with morbidity [2,3]. Published surgical series report 15 to 30% rates of new or worsening visual deficits following operation. The need to achieve tumor control while preserving visual function has prompted the development of specific radiation delivery techniques [4].

Stereotactic radiosurgery is a well-established treatment modality for skull base meningiomas [5,6]. However, anterior visual pathways bespeak high fraction sensitivity, with the optic nerve and chiasm characterized by a low α/β ratio (approximately 1–2 Gy), that translates into a high probability of delayed radiation injury, as a function of fraction dose [7].

Single-fraction radiosurgery protocols mandate a maximum dose of 10–12 Gy to the optic apparatus, that often limits, in cases of large lesions or tumors in close contact with or even encasing visual structures, an adequate target coverage [8,9,10]. Accumulated clinical evidence demonstrates that the incidence of optic neuropathy remains below 1–3% when established dosimetric constraints are respected: 12 Gy in single-fraction, 20 Gy in three fractions, or 25 Gy in five fractions to the anterior visual pathway [11]. Fractionated treatment paradigms significantly reduce visual morbidity compared to single-session approaches, in case of lesions involving, or close to, the optic apparatus.

Conventional Gamma Knife radiosurgery uses the invasive Leksell stereotactic frame for rigid cranial immobilization [12]. The system guarantees high geometric accuracy (submillimetric precision) in single-session radiosurgery, but has obvious constraints for fractionated treatment protocols. Wearing a frame across multiple consecutive sessions or daily serial frame application and removal is impractical, generates significant patient discomfort and requires multiple RM acquisitions This adversely impacts treatment compliance and tolerability [13].

The Leksell Gamma Knife Icon system represents a revolution in stereotactic radiosurgery technology, integrating frameless thermoplastic mask-based immobilization with high-definition cone-beam computed tomography (CBCT) for stability of treatment plan geometry between sessions and intrafractional motion surveillance [14]. Precise patient repositioning and accuracy equivalent to frame-based techniques are reached without invasive fixation. The CBCT-based workflow incorporates highly automated patient positioning with six-degrees-of-freedom corrections and continuous motion tracking throughout beam delivery, eliminating practical constraints and patient tolerance limitations encountered in frame-based fractionated Gamma Knife therapy [15].

Given the relatively recent introduction of these technological innovations, evidence regarding long-term clinical outcomes following frameless fractionated Gamma Knife (GK) radiosurgery for perioptic meningiomas is limited [16].

Few papers are found in the literature describing Icon GK fractionated therapy for lesions enclosing or abutting the anterior visual pathways: all of them report series with limited patient accrual, or short clinical and instrumental follow-up.

The present investigation explores a clinical series of multi-fraction Gamma Knife Icon radiosurgery (GKRS) specifically dedicated to perioptic meningiomas, including extended follow-up and comprehensive analysis of tumor control rates, visual functional outcomes, treatment-associated toxicity, and overall therapeutic safety profile.

## 2. Materials and Methods

We performed a retrospective observational study subject to prior institutional board approval. We included all patients who, from September 2017 to December 2022, underwent hypofractionated Gamma Knife treatment with the Icon system using a thermoplastic mask for perioptic meningiomas (in contact or within 3 mm distance). For the purposes of this study, “perioptic meningioma” was defined as any meningioma whose growth—regardless of primary dural attachment site—results in direct anatomical proximity to (3 mm), or radiological contact with, the optic nerves or optic chiasm, thereby placing the anterior visual pathway at dosimetric risk during radiosurgical treatment planning. Therefore, our sample included three different GKRS scenarios: (1) upfront GKRS as primary therapy, (2) adjuvant GKRS for post-surgical residual disease, and (3) salvage GKRS for tumor recurrence.

All patients, selected by a neurosurgeon, were then treated under the care of a multidisciplinary team (neurosurgeon, radiation oncologist, and medical physicist).

Regarding the multidisciplinary process, treatment decisions were made on an individualized basis within a dedicated neuro-oncology tumor board, involving neurosurgeons, radiation oncologists, neuroradiologists, and ophthalmologists/neuro-ophthalmologists. For each patient, the decision to proceed with radiosurgery—rather than active surveillance—was based on a structured assessment incorporating the following criteria: documented radiological tumor growth on serial imaging, onset or progression of neurological or visual symptoms, patient age and comorbidity profile, and patient preference following detailed informed consent. Each patient was analyzed per: age, sex, lesion location, volume, previous surgery, and histology if available. Pre-treatment volumes were calculated using the Leksell Gamma Plan Treatment Planning System. The decision to use either 3 or 5 fractions was driven by the dose delivered to the critical organs (optic nerve and optic chiasm) at risk and the need to maintain appropriate dose constraints. Specifically, a 3-fraction regimen was preferred whenever dose constraints to critical structures could be adequately met; conversely, when the dose to organs at risk exceeded 18–20 Gy, a 5-fraction schedule was selected in order to ensure compliance with accepted tolerance thresholds. Clinical features as well as any pre-treatment neurological deficit, including visual disturbances (both visual acuity and visual field defects), oculomotor disorders, and trigeminal involvement, were recorded. Regarding GK treatment parameters, we included marginal dose (Gy), number of fractions, coverage, Paddick index (PI). In addition, we collected the maximum dose to 1 mm^3^ (Gy) on both the optic chiasm and optic nerves.

### 2.1. Exclusion Criteria

- Patients under 18 years of age;
- WHO grade II meningiomas;
- Patients with clinical and radiological follow-up shorter than 2 years;
- Patients previously treated with brain radiotherapy.

### 2.2. Workflow with GK Icon System

Target definition was drawn on a 1.5 Tesla MRI (GE, Sigma Explorer, Chicago, IL, USA), volumetric axial T1 sequence without and with contrast, and axial T2, performed on the first day of treatment or shortly before. The procedure started with modeling a thermoplastic face mask. Cone-beam CT (CBCT) images were fused with the RM treatment plan images and defined as treatment planning images (reference CBCT). Daily CBCT obtained on each fraction day was co-registered with the reference CBCT. Position error (treatment offset) was displayed and, after correction and approval, transferred to the treatment machine.

On every treatment day, mask was repositioned and a pre-treatment CBCT captured. After correction of offsets and treatment approval, a reflective optical marker was placed on the patient’s nose tip to serve as an anatomical reference point. Once treatment is started, the patient’s position is monitored by an infrared camera that detects displacement by tracking the nose tip. If the motion detected exceeds a preset threshold (default of 1.0 mm), the treatment is halted.

### 2.3. Outcome

Clinical and radiological data were collected for each patient. The distinct innovation of this study is patient repositioning using a thermoplastic mask without an invasive stereotactic frame for GK treatment. Therefore, the primary objective of this study was to verify the safety of the procedure over multiple fractions based on mask immobilization, focused on potential adverse effects on the optic pathways, specifically regarding RION [17]. As the primary endpoint, we considered the clinical tumor control defined by using the clinical follow-up assessment categorized as: improved, stable, or worsened. Variables used to define improved/worsened groups were visual function, visual field, oculomotor function, and trigeminal symptoms. Improved or worsened was defined in terms of these 4 variables: an improvement/worsening in at least one of these variables was defined as “improved/worsened patient”. For patients with pre-GK symptoms, we recorded any change at clinical follow-up. Studies with post-contrast MRI were routinely performed 6 months and one year after GK, and then every year thereafter. Clinical follow-up consisted of visual field testing and ophthalmological examinations performed annually. The secondary endpoint was the radiological tumor control. Radiological follow-up included the delineation of volumes of interest on 3D T1 post-contrast volumetric sequences delineated on Leksell Gamma Plan from the most recent available radiological examination. We considered lesions to be reduced when there was a volume change of 25% or greater [18].

### 2.4. Statistical Analysis

Descriptive statistics per patient were reported as mean ± standard deviation (SD) for continuous variables and as frequency and percentage (%) or rate for categorial variables. Group comparisons were assessed using Student’s *t*-test or the non-parametric Mann–Whitney test for continuous variables, and the Chi-squared test or tests for proportions for categorical variables. The variables that were statistically different between the groups under analysis were concurrently considered in multiple logistic models to assess their potential predictor for discriminating between the groups. The results of these models were presented by odds ratios (ORs) and 95% CI. Statistical analyses were performed using the R statistical software (URL: https://www.R-project.org/; version 4.4.0).

## 3. Results

Between September 2017 and December 2022, a total of 100 patients were enrolled, including 80 females and 20 males. Table 1 summarizes the main demographic characteristics. The mean age was 61.7 years (range 35–84). Of these patients, 32 had undergone previous surgery with histological confirmation of WHO grade I meningioma. For the remaining 68 patients, the diagnosis was radiological. Among patients with previous surgery, treatment was performed as adjuvant therapy in 9 cases, while in 23, it was administered as salvage therapy for recurrence. The most common locations of the treated meningiomas were: cavernous sinus (35 pts), clinoid (17 pts), sphenoid wing (14 pts), olfactory groove (12 pts), and tuberculum sellae (10 pts). Less frequently involved locations included spheno-orbital (4 pts), petroclival (2 pts), and optic nerve sheath meningiomas (2 pts). At the time of treatment, 35 patients presented with neurological symptoms, including 19 with visual disturbances, 22 with visual field impairment, and 17 with ocular motility disorders (cranial nerve III, IV, and VI deficits). Nine patients experienced trigeminal symptoms. Ten patients simultaneously presented with more than one symptom. None of the enrolled patients received concomitant steroid therapy at the time of GK treatment.

### 3.1. Radiosurgical Parameters

The mean lesion volume at GK treatment was 6.92 mL (SD 6.2, range 0.1–31.7) with a median of 5.6 mL. For each patient subjected to GKRS, immobilization was achieved utilizing the GK Icon thermoplastic mask.

In most cases (89%), we performed five-fraction Icon GK treatment with a marginal dose of 25 Gy (5 Gy/fraction); in 10 patients (10%) we performed 3-day GK with a marginal dose of 21 Gy (7 Gy/fraction), and in only 1 patient (1%), four-fraction treatment was delivered (5 Gy/fraction) with a marginal dose of 20 Gy. In this single case, a four-fraction schedule was adopted purely for logistical reasons related to the patient’s availability. The mean dose to 1 mm^3^ of the optic nerve was 17.5 Gy (SD 4.4, range 6.0–27.0, median 18.4 Gy), while the mean dose to 1 mm^3^ of the optic chiasm was 13.8 Gy (SD 6.17, range 3.8–35.5, median 13.5 Gy).

### 3.2. Clinical Follow-Up

Clinical follow-up was available for 99 patients (1 patient lost to follow-up), with a mean follow-up of 45.3 months (SD 18.2, range 6–92 months, median 39 months). Of the 99 patients with available clinical follow-up, visual deterioration was recorded in only one patient. At the time of the study, among these 99 patients, 3 had died from causes unrelated to radiosurgical treatment of the meningioma (2 patients from COVID-19 and 1 patient from lung cancer). Nevertheless, clinical and radiological follow-up exceeding 24 months was available for these patients.

Clinical outcomes demonstrated clinical stability in 91 patients (92%), improvement in 5 patients (5%), and clinical deterioration in 3 patients (3%). Among the 35 symptomatic patients at baseline, 3 reported worsening symptoms (14% of symptomatic patients), while 5 showed improvement (9% of symptomatic patients). Among those with deterioration, one patient (1%) presented with concurrent tumor regrowth requiring surgical intervention, and one patient experienced worsening of trigeminal dysesthesias. Visual pathway deterioration occurred in only 1 of 99 cases (1%), notably in a patient who had presented with pre-existing visual disturbances prior to treatment. None of the 65 asymptomatic patients developed new symptoms and no instances of acute toxicity were observed. In no case did blindness occur.

Only one patient with an olfactory groove meningioma developed perilesional edema, which was treated with steroid therapy, resulting in symptom regression characterized primarily by headache. No patient experienced post-treatment seizures in the follow-up period.

Table 2 compares characteristics between patients who showed clinical improvement (5, 5%) after GKRS and “other” (those remained either clinically stable or deteriorated (94,95%). Gender and age were similar between groups, with no significant differences (*p* = 1.0 and *p* = 0.829 respectively). Tumor location distribution was comparable (*p* = 0.812), though the improved group had a higher proportion of cavernous sinus tumors (60% vs. 34%). Mean tumor volume was smaller in the improved group (4.6 cc vs. 7.1 cc), though this did not reach statistical significance (*p* = 0.354). Dose to the optic chiasm was similar between groups (*p* = 0.467). Patients who improved had a significantly higher rate of baseline visual symptoms (80% vs. 33%, *p* = 0.046). Similarly, 80% of improved patients had baseline visual field deficits compared to only 19% in the stable group (*p* = 0.008). Trigeminal dysesthesias and cranial nerve deficits showed no significant differences (*p* = 0.942).

Table 2 summarizes the clinical outcome of the patients, specifically comparing the characteristics of patients who experienced improvement of pre-treatment symptoms compared to the “other” group (stable and worsened). In total, 94.73% and 90.9% of improved patients had pre-treatment visual and visual field deficits, respectively, compared to only 5.27% and 9.1% in the stable group. Tumor location distribution was not different between the two groups (*p* = 0.812), though the improved group had a higher proportion of cavernous sinus tumors (60% vs. 34.1%).

Table 3 summarizes the characteristics of patients who experienced clinical worsening (N = 3) with those “other” (who remained clinically stable or improved) following GKRS. None of the analyzed variables reached statistical significance in predicting clinical deterioration. Although not statistically significant (*p* = 0.217), worsened patients had notably larger mean volumes (12.6 cm^3^ vs. 6.77 cm^3^). In dosimetric parameters, no difference was observed, both in dose delivered to 0.001 cc optic nerve (*p* = 0.707) and dose delivered to 0.001 cc optic chiasm (*p* = 0.878). Baseline symptoms showed no association with worsening. Follow-up duration was comparable between groups.

### 3.3. Radiological Follow-Up

Radiological follow-up with serial contrast-enhanced MR was available in 98 patients (2 patients lost to follow-up), with a mean follow-up of 41.2 months (SD 19.4, range 24–92 months, median 36 months), as shown in Table 4.

Overall tumor control was achieved in 97 of 98 (99%) patients with radiological follow-up. Radiological outcomes showed stable disease in 76 patients (78%), volume reduction in 21 patients (21%), and volume increase in 1 patient (1%). The single patient who demonstrated tumor volume increase at follow-up was subsequently treated with surgery. Cavernous sinus location is much more common in the “reduced” group (62% vs. 28%). Coverage (~98–99%) and Paddick index (PI ~0.70) were excellent and similar between groups. Marginal dose was slightly higher in “reduced” group (25 Gy vs. 24.3 Gy, *p* = 0.026—statistically significant).

Considering both the significant (at *p*-value < 0.1) variables marginal dose (Gy) and radiological fu duration in a multiple logistic regression model, their association with the two groups is confirmed: both variables had a significant odds ratio (OR = 12.1, 95% CI [1.5, 97.8] for marginal dose and OR = 1.1, 95% CI [1.01, 1.3]).

### 3.4. Prior Surgery

Table 5 compares outcomes between patients who underwent GK treatment as primary therapy (N = 67, 68%) versus those who received GK for residual or recurrent disease following surgery (N = 31, 32%). Tumor location showed a statistically significant correlation with the treatment group (*p* = 0.006). In particular, cavernous sinus meningiomas were predominantly managed with primary radiosurgery (45% vs. 13%), reflecting the surgical challenges and high morbidity associated with resection at this location. Sphenoid wing and spheno-orbital meningiomas were more frequently treated with surgery first (26% and 13% in the surgical group vs. 9% and 0% in the primary GK group). Radiological outcomes and tumor response patterns also differed significantly between groups (*p* = 0.035): tumor reduction was achieved in 28% of patients in the primary GK group compared with 6.5% in the post-surgical GK group, representing an approximately 4-fold higher reduction rate with primary radiosurgery.

Considering both significant variables—location and duration of radiological follow-up—in a multiple logistic regression model, their association with the two groups was confirmed. Both variables showed significant odds ratios (OR = 2.1, 95% CI [1.2, 4.8] for location; OR = 1.3, 95% CI [1.1, 1.6] for follow-up duration). These results indicate that tumor location significantly influences the decision to undergo GK treatment as a primary therapy, and that GK treatment is associated with a higher likelihood of achieving stable and/or improved radiological follow-up outcomes.

## 4. Discussion

### 4.1. Clinical Follow-Up

Radiation-induced optic neuropathy (RION) represents the most significant dose-limiting threat in managing perioptic meningiomas. The radiobiological advantage of exploiting differential α/β ratios between tumor and optic structures, combined with the ability to deliver higher biologically effective doses while respecting normal tissue tolerance, makes hypofractionated radiosurgery the preferred treatment approach for meningiomas involving or abutting the optic apparatus [7].The shift from single-fraction stereotactic radiosurgery to hypofractionated stereotactic radiosurgery has been driven by radiobiological principles and clinical evidence demonstrating superior safety profiles [19]. However, it should be noted that the linear-quadratic (LQ) model presents well-recognized limitations when applied to radiosurgery, where large doses per fraction fall outside the dose range for which the model was originally validated [20]. At ablative dose levels, the LQ framework tends to underestimate biological effectiveness, as vascular and indirect cell-kill mechanisms—not captured by the model—become increasingly relevant [21]. Furthermore, the α/β ratio commonly assigned to the optic pathways (2–3 Gy) carries substantial uncertainty, being largely extrapolated from conventional fractionation data rather than directly derived from high-dose clinical or experimental evidence specific to this tissue. Given the narrow tolerance window of the optic nerves and chiasm, even modest inaccuracies in this parameter may introduce non-negligible errors in biologically effective dose (BED) estimates. These considerations warrant careful interpretation of radiobiological calculations in this context, and LQ-derived estimates should be regarded as approximations rather than precise biological predictors [22].

The convergence of multiple institutional experiences and comparative studies supports multi-fraction as an optimal balance between efficacy and safety, with strict adherence to maximum optic pathway doses ≤25 Gy being critical for minimizing RION risk. RION rates have decreased from 8 to 20% with single-fraction approaches to <2–5% with hypofractionation, while maintaining excellent local control rates of 90–95% [23,24]. A recent meta-analysis reports a complication rate of approximately 9.6% (visual decline) for single-fraction SRS and 5.1% for hSRA [25]; these results were pooled from several centers, including different radiosurgery techniques such as Gamma Knife, CyberKnife, and LINAC-based systems. Our study represents one of the largest series of fractionated Gamma Knife treatments using a non-invasive fixation system, with the thermoplastic mask, and this also represents an undeniable advantage for most patients. There are fewer than ten studies specifically dedicated to the use of Icon GK (frameless) for perioptic meningiomas with fractionated GK in the current literature, almost all retrospective and with small cohorts. The most recent include 24 patients with HF-GKRS (75% with tumors near the optic pathway), achieving a 1-year and 3-year PFS of 100% and 92%, respectively, with a 3-year adverse radiation effect rate of only 9% and 50% of patients showing tumor reduction [26]. Another recent multicenter study of 34 patients (40 tumors) treated found no significant visual deterioration in 16 tumors within 3 mm of the optic apparatus, with a 5-year tumor progression of 7.7% for low-grade meningiomas [27].

Despite the absence of an invasive head fixation system, in this cohort we confirmed the excellent safety profile with less than 3% clinical worsening. The incidence of visual deterioration in our series is modestly lower (1,01%) than that recently described in a retrospective observational study, wherein complication rates were reported as 7.9% for optic neuropathy and 2,8% of severe adverse radio-induced events (grade 3–4 toxicity) [28,29]. Regarding predictive factors for clinical worsening, no statistically significant correlations were observed with any of the dosimetric or radiological parameters, although tumor volume could represent a potential risk factor: the trend toward larger volumes in worsened patients (12.6 vs. 6.77 cm^3^) warrants attention in treatment planning, though more data are needed for definitive conclusions. This nearly two-fold difference suggests that larger tumor size might represent a risk factor, though the small sample does not allow for definitive conclusions. No correlation between dose to optic pathway and worsening were observed, as well as the dose to 1 mm^3^ to optic pathway. Our results suggest that radiation dose alone did not predict clinical worsening when applied within standard safety constraints [11]. Moreover, the absence of significant predictors suggests that clinical worsening may be multifactorial or related to unmeasured variables such as individual radiosensitivity or tumor biology. The very small number of worsened patients (N = 3) severely limits statistical power and the ability to identify significant risk factors. A larger cohort or meta-analysis would be needed to definitively identify predictors of clinical deterioration. Moreover, we acknowledge that defining clinical improvement or worsening based on a change in any one of the four assessed variables may represent a broad criterion, potentially leading to an overestimation of clinically meaningful changes. Analysis of the characteristics of the five patients demonstrating clinical improvement revealed that the most notable finding was the significantly higher likelihood of baseline visual symptoms (including visual disturbances and visual field defects) in this cohort. Although this observation may seem self-evident, it indicates that symptomatic patients at presentation may benefit from Gamma Knife radiosurgery with the potential for symptom resolution, while asymptomatic or oligosymptomatic patients typically maintain a stable clinical course. Concerning the treatment of asymptomatic cases, we acknowledge that a subset of patients in this series was treated in the absence of overt symptoms. This reflects a deliberate institutional philosophy grounded in the recognition that perioptic meningiomas carry a significant risk of delayed but potentially irreversible visual deterioration, and that early intervention—prior to the onset of symptomatic compromise—may afford superior visual preservation outcomes compared to deferred treatment.

### 4.2. Radiological Follow-Up

Our series demonstrated excellent radiological tumor control (99%; 97 of 98 patients), with a single case of radiological progression requiring salvage surgery. These results compare favorably with the meta-analysis by Peters et al., which included 865 perioptic meningioma cases and reported overall tumor control rates of 95.1% for single-fraction stereotactic radiosurgery and 95.6% for hypofractionated treatment, confirming near-equivalent efficacy between treatment paradigms. Additionally, tumor volumetric reduction was observed in 21 patients.

A notable, though not statistically significant, difference was observed in tumor location distribution. Cavernous sinus meningiomas were notably over-represented in the reduction group (61.9% vs. 27.8%), suggesting that tumors in this location may be more likely to show volumetric reduction following treatment. This trend suggests that certain locations may be more amenable to volumetric response, possibly related to tumor biology or blood supply. Regarding dosimetric parameters, the only statistically significant difference between groups was the marginal dose, slightly higher in the volumetric reduction group (mean 25.0 Gy vs. 24.3 Gy, *p* = 0.026). These preliminary observations suggest that delivering the full prescribed dose of 25 Gy is associated with a higher probability of tumor shrinkage. While a statistically significant difference was observed, the absolute dose variation is unlikely to translate into a meaningful clinical distinction, and that this finding should therefore be interpreted with caution. Moreover, patients in the reduced volume group have a trend toward longer radiological follow-up (49.5 vs. 38.9 months, *p* = 0.065), possibly because they may have allowed more time for volumetric response to manifest. These data suggest that tumor volume reduction following multi-fraction GKRS may be influenced by marginal dose and potentially by tumor location, particularly cavernous sinus meningiomas. Importantly, the absence of differences in doses to critical structures indicates that tumor reduction was achieved without compromising radiation safety to the anterior visual pathways.

### 4.3. Prior Surgery

Table 5 compares outcomes between patients who underwent hypofractionated GKRS as primary therapy versus those who received hypofractionated GKRS for residual or recurrent disease after surgery. Tumor response patterns differed substantially between treatment approaches, with radiological volumetric reduction observed in 28.4% of patients undergoing upfront GKRS compared to only 6.5% of those receiving adjuvant GKRS after surgical intervention. Primary radiosurgery is significantly more likely to induce tumor shrinkage than treating post-surgical residual/recurrent disease. This may reflect both post-surgical fibrosis/scarring affecting radiosensitivity and better target definition and coverage in unoperated tumor. These results corroborate previous reports demonstrating that upfront radiosurgery represents the only independent prognostic factor associated with enhanced tumor control [28]. Despite lower reduction rates, post-surgical GK provides excellent tumor control (93.5% stable, 0% progression) and clinical outcomes comparable to primary treatment. The data could support a location-based treatment algorithm: primary radiosurgery for cavernous sinus and other surgically challenging locations, with surgery reserved for accessible lesions. Post-surgical patients should be counseled that stability rather than shrinkage is the expected outcome, though clinical efficacy remains excellent.

### 4.4. Study Limitations

This study has several limitations that should be acknowledged. First, its retrospective and single-center design inherently introduces selection bias and limits the generalizability of the findings to broader patient populations. Second, the fractionation protocols employed were not evenly distributed, with a marked predominance of five-fraction regimens and the absence of a true control group, making direct comparisons between schedules difficult to draw. Moreover, the number of cases showing clinical or radiological deterioration was relatively small, resulting in underpowered and unbalanced comparison groups that preclude robust statistical conclusions regarding predictors of worsening outcomes. Finally, the present findings may not be generalizable across all radiosurgical platforms (LINAC, CK, GK, etc.) and the prospective comparative studies would be required to establish the superiority or equivalence of any single modality in this anatomical location.

## 5. Conclusions

This study demonstrates that five-fraction Gamma Knife Icon radiosurgery represents a safe, effective, and well-tolerated treatment approach for perioptic meningiomas. The frameless thermoplastic mask system eliminates the need for invasive frame placement while maintaining high targeting precision, thereby improving both feasibility and patient compliance. Fractionated GK with the Icon system uniquely combines the dosimetric advantages of Gamma Knife—including sharp dose gradients and reduced integral body dose—with the radiobiological benefits of fractionated delivery through a non-invasive immobilization system. This technological advancement overcomes historical limitations of single-fraction Gamma Knife radiosurgery, extending treatment eligibility to perioptic meningiomas previously considered suboptimal candidates due to tumor size or proximity to critical neurovascular structures.

## Figures and Tables

**Table 1:** Summary table of the main demographic and dosimetric characteristics of the sample analyzed: 100 patients treated with GK Icon using a thermoplastic mask for meningiomas in proximity to the optic pathways.

	(N = 100)
**Gender**	
F	80 (80%)
M	20 (20%)
**Age**	
Mean (SD)	61.7 (11.7)
Median [Min, Max]	61.0 [35.0, 84.0]
**Location**	
sphenoid wing	14 (14%)
apex petrous bone	1 (1%)
clinoid	17 (17%)
clivus	1 (1%)
olfactory groove	12 (12%)
optic nerve sheath	3 (3%)
petroclival	2 (2%)
cavernous sinus	35 (35%)
spheno-orbital	4 (4%)
tentorial	1 (1%)
tuberculum	10 (10%)
**Previous surgery**	32 (32%)
Adjuvant GKRS	9 (28%)
Salvage GKRS	23 (72%)
**Volume cm^3^**	
Mean (SD)	6.9 (6.2)
Median [Min, Max]	5.62 [0.12–31.7]
**Visual disorders at GKRS**	
yes	19 (19%)
no	81 (81%)
**Visual field disorders at GKRS**	
no	78 (78%)
yes	22 (22%)
**Trigeminal dysesthesias at GKRS**	
no	91 (91%)
yes	9 (9%)
**III-IV-VI cn deficits at GKRS**	
no	83 (83%)
yes	17 (17%)
**Mean dose to optic nerve**	
Mean (SD)	9.0 (3.3)
Median [Min, Max]	9.0 [1.8, 17.8]
**Coverage**	
Mean (SD)	1.0 (1.3)
Median [Min, Max]	25.0 [20.0, 25.0]
**PI**	
Mean (SD)	0.7 (0.1)
Median [Min, Max]	0.7 [0.2, 0.9]
**Number of fractions**	
3	10 (10%)
4	1 (1%)
5	89 (89%)
**Clinical fu (months)**	
Mean (SD)	45.3 (18.2)
Median [Min, Max]	39.0 [6.0, 92.0]
Missing	1 (1%)
**Clinical fu**	
Stable	91 (92%)
Improved	5 (5%)
Worsened	3 (3%)
Missing	1 (1%)
**Radiological fu**	
Missing	2 (2%)
Increased	1 (1%)
Reduced	21 (21%)
Stable	76 (76%)
**Radiological fu (months)**	
Mean (SD)	41.2 (19.4)
Median [Min, Max]	36.0 [24.0–92.0]
Missing	2 (2%)

**Table 2:** The table summarizes the clinical outcome of the patients, specifically comparing the characteristics of patients who experienced improvement of pre-treatment symptoms compared to the “other” group (stable and worsened).

CLINICAL FOLLOW-UP	Other	Improved	*p*-Value
	(N = 94)	(N = 5)	
**Gender**			
F	75 (80%)	4	1.000
M	19 (20%)	1	
**Age**			
Mean (SD)	61.6 (11.9)	61.0 (8.1)	0.829
Median [Min, Max]	61.5 [35.0, 84.0]	59.0 [54.0, 75.0]	
**Location**			
sphenoid wing	14 (15%)	0 (0%)	0.812
apex petrous bone	1 (1%)	0 (0%)	
clinoid	17 (18%)	0 (0%)	
clivus	1 (1%)	0 (0%)	
olfactory groove	11 (12%)	0 (0%)	
optic nerve sheath	2 (2%)	1 (20%)	
petroclival	2 (2%)	0 (0%)	
cavernous sinus	32 (34%)	3 (60%)	
spheno-orbital	4 (4%)	0 (0%)	
tentorial	1 (1%)	0 (0%)	
tuberculum	9 (10%)	1 (20%)	
**Pre-GKRS visual disorders**			
Yes (19 patients)	18 (95%) *	1 (5%) *	**0.046**
**Pre-GKRS visual field disorders**			
Yes (22 patients)	20 (91%) *	2 (9%) *	**0.008**
**Pre-GKRS trigeminal dysesthesia**			
Yes (9 patients)	8 (89%) *	1 (11%) *	0.942
**Pre-GKRS III-IV-VI CN deficits**			
Yes (17 patients)	16 (94%) *	1 (6%) *	0.435
**Number of fractions**			
3	9 (10%)	0 (0%)	0.744
4	1 (1%)	0 (0%)	
5	84 (89%)	5 (100%)	
**Volume (cm^3^)**			
Mean (SD)	7.1 (6.3)	4.6 (4.8)	0.354
Median [Min, Max]	5.8 [0.1, 31.7]	2.7 [0.2, 11.9]	
**Clinical fu (months)**			
Mean (SD)	45.0 (18.0)	51.6 (22.9)	0.443
Median [Min, Max]	39.0 [6.0, 92.0]	53.0 [24.0, 84.0]	
**Pre-GK surgery**			
no	63 (67%)	4 (80%)	0.938
yes	30 (32%)	1 (20%)	
**PI**			
Mean (SD)	0.7 (0.12)	0.6 (0.16)	0.227
Median [Min, Max]	0.7 [0.2, 0.9]	0.7 [0.4, 0.8]	
**Coverage**			
Mean (SD)	1.0 (0.03)	1.0 (0.1)	0.744
Median [Min, Max]	1.0 [0.8, 1.0]	1.0 [0.4, 0.8]	

* Row percentage. Bolded *p*-values indicate statistically significant results (*p* < 0.05).

**Table 3:** The table compares patients who remained clinically stable (N = 96) with those who experienced clinical worsening (N = 3) following GK. The excellent safety profile is immediately apparent, with only 3% of patients (3/99) showing clinical deterioration.

CLINICAL FOLLOW-UP	Other	Worsened	*p*-Value
	(N = 96)	(N = 3)	
**Gender**			
F	78 (81%)	1 (33%)	0.192
M	18 (19%)	2 (67%)	
**Age**			
Mean (SD)	61.4 (11.7)	66.0 (12.3)	0.513
Median [Min, Max]	61.0 [35.0, 84.0]	61.0 [57.0, 80.0]	
**Location**			
sphenoid wing	14 (15%)	0 (0%)	0.666
apex petrous bone	1 (1%)	0 (0%)	
clinoid	16 (17%)	1 (33%)	
clivus	1 (1.0%)	0 (0%)	
olfactory groove	11 (11%)	0 (0%)	
optic nerve sheath	3 (3%)	0 (0%)	
petroclival	2 (2%)	0 (0%)	
cavernous sinus	34 (35%)	1 (33%)	
spheno-orbital	3 (3%)	1 (33%)	
tentorial	1 (1%)	0 (0%)	
tuberculum	10 (10%)	0 (0%)	
**PI**			
Mean (SD)	0.7 (0.1)		
Median [Min, Max]	0.7 (0.1)		
**Dose delivered to 1 mm^3^ cc optic nerve**			
Mean (SD)	17.6 (4.3)	16.8 (4.1)	0.707
Median [Min, Max]	18.5 [6.0, 27.0]	17.1 [12.6, 20.7]	
**Dose delivered to 1 mm^3^ optic chiasm**			
Mean (SD)	13.8 (6.2)	14.5 (6.0)	0.878
Median [Min, Max]	13.6 [3.8, 35.5]	13.3 [9.1, 21.0]	
**D max to optic chiasm**			
Mean (SD)	15.8 (6.7)	15.6 (6.8)	0.898
Median [Min, Max]	16.5 [4.10, 29.7]	14.3 [9.5, 22.9]	
**D max to optic nerve**			
Mean (SD)	20.0 (5.0)	19.1 (4.2)	0.624
Median [Min, Max]	21.2 [7.0, 1.0]	19.5 [14.7, 23.0]	
**Mean dose to optic chiasm**			
Mean (SD)	8.6 (3.9)	9.6 (3.2)	0.668
Median [Min, Max]	8.6 [2.0,18.1]	8.5 [7.2, 13.2]	
**Mean dose to optic nerve**			
Mean (SD)	9.1 (3.3)	8.7 (4.7)	1.000
Median [Min, Max]	9.0 [1.8, 17.8]	9.6 [3.6, 12.9]	
**Pre-GKRS visual disorders**			
Yes	18 (95%) *	1 (5%) *	1.000
No	80 (100%) *	0 (0%) *	
**Pre-GKRS visual field disorders**			
Yes	21 (96%) *	1 (4%) *	1.000
No	77 (100%) *	0 (0%) *	
**Pre-GKRS trigeminal dysesthesias**			
Yes	8 (89%) *	1 (11%) *	0.643
No	90 (100%) *	0 (0%) *	
**Pre-GKRS III-IV-VI cn deficits**			
Yes	17 (100%) *	0 (%) *	0.981
No	82 (100%) *	0 (0%) *	
**Number of fractions**			
3	9 (9%)	0 (0%)	0.840
4	1 (1%)	0 (0%)	
5	86 (90%)	3 (100%)	
**Volume at GK (cm^3^)**			
Mean (SD)	6.8 (6.1)	12.6 (10.8)	0.217
Median [Min, Max]	5.6 [0.1, 31.7]	10.0 [3.2, 24.5]	

*: Row percentage.

**Table 4:** This table compares patients who achieved tumor volume reduction (N = 21, 21%) with those showing other radiological outcomes (stable or progressed, N = 79, 79%) following GK treatment. Patients with tumor reduction received a slightly higher mean marginal dose (25.0 Gy vs. 24.3 Gy); while the absolute difference appears small (~0.7 Gy), it reached statistical significance (*p* = 0.026). The mean volumes were nearly identical (6.9 vs. 7.0 cm^3^), indicating baseline size does not predict shrinkage.

RADIOLOGICAL FOLLOW-UP	Other	Reduced	*p*-Value
	(N = 77)	(N = 21)	
**Gender**			
F	64 (81%)	16 (76%)	0.854
M	13 (19%)	5 (24%)	
**Age**			
Mean (SD)	62.1 (12.1)	60.3 (10.4)	0.482
Median [Min, Max]	62.0 [35.0, 84.0]	59.0 [41.0, 80.0]	
**Location**			
sphenoid wing	14 (18%)	0 (0%)	0.264
apex petrous bone	1 (1%)	0 (0%)	
clinoid	12 (15%)	5 (24%)	
clivus	1 (1%)	0 (0%)	
olfactory groove	10 (13%)	2 (10%)	
optic nerve sheath	3 (4%)	0 (0%)	
petroclival	2 (3%)	0 (0%)	
cavernous sinus	22 (28%)	13 (63%)	
spheno-orbital	4 (5%)	0 (0%)	
tentorial	1 (1%)	0 (0%)	
tuberculum	9 (11%)	1 (5%)	
**Dose to 1 mm^3^ cc optic nerve**			
Mean (SD)	17.3 (4.6)	18.3 (3.2)	0.451
Median [Min, Max]	18.0 [6.0, 27.0]	18.5 [9.5, 23.0]	
**Dose to 1 mm^3^ optic chiasm**			
Mean (SD)	13.3 (5.9)	15.6 (7.1)	0.250
Median [Min, Max]	13.5 [3.8, 24.5]	15.0 [4.0, 35.5]	
**D max to optic chiasm**			
Mean (SD)	19.5 (5.3)	20.9 (3.9)	0.401
Median [Min, Max]	20.7 [7.0, 31.5]	21.1 [9.8, 28.4]	
**D max to optic nerve**			
Mean (SD)	8.86 (3.27)	9.5 (3.6)	0.585
Median [Min, Max]	8.70 [1.8, 17.8]	9.0 [3.1, 17.6]	
**Marginal dose (Gy)**			
Mean (SD)	24.3 (1.5)	25.0 (0)	**0.026**
Median [Min, Max]	25.0 [20.0, 25.0]	25.0 [25.0, 25.0]	
**Coverage**			
Mean (SD)	0.98 (0.03)	0.99 (0.01)	0.871
Median [Min, Max]	0.99 [0.77, 1.00]	0.99 [0.97, 1.00]	
**PI**			
Mean (SD)	0.70 (0.13)	0.70 (0.10)	0.809
Median [Min, Max]	0.72 [0.18, 0.91]	0.71 [0.39, 0.81]	
**Pre-GKRS visual disorders**			
no	67 (87%)	12 (57%)	0.658
yes	10 (13%)	9 (43%)	
**Pre-GKRS visual field disorders**			
no	63 (80%)	15 (71%)	0.602
yes	16 (20%)	6 (29%)	
**Pre-GKRS trigeminal paresthesia**			
no	74 (94%)	17 (81%)	0.167
yes	5 (6%)	4 (19%)	
**Pre-GKRS cranial nerve deficit**			
no	68 (86%)	15 (71%)	0.207
yes	11 (14%)	6 (29%)	
**Number of fraction**			
3	10 (13%)	0 (0%)	0.193
4	1 (1%)	0 (0%)	
5	68 (86%)	21 (100%)	
**Volume (cm^3^)**			
Mean (SD)	6.9 (6.5)	7.0 (5.5)	0.639
Median [Min, Max]	5.6 [0.1, 31.7]	5.7 [0.15, 22.3]	
**Radiological follow-up (months)**			
Mean (SD)	38.9 (17.5)	49.5 (23.9)	0.065
Median [Min, Max]	36.0 [0, 92.0]	54.0 [12.0, 84.0]	

Bolded *p*-values indicate statistically significant results (*p* < 0.05).

**Table 5:** This table compares outcomes between patients who underwent GK treatment upfront, as primary therapy (N = 67, 68%), versus those who received GK for residual or recurrent disease after surgery (N = 31, 32%). Radiological follow-up showed significant differences (*p* = 0.035) between patients with prior surgery and those without prior surgery. The non-surgery group showed higher rates of tumor reduction (28%) compared to the prior surgery group (7%).

Pre-GKRS Surgery	No	Yes	*p*-Value
	(N = 67)	(N = 31)	
**Gender**			
F	55 (82%)	24 (77%)	0.788
M	12 (18%)	7 (23%)	
**Age**			
Mean (SD)	61.9 (11.5)	60.8 (12.4)	0.649
Median [Min, Max]	61.0 [35.0, 84.0]	60.0 [37.0, 83.0]	
**Location**			
sphenoid wing	6 (9%)	8 (26%)	
apex petrous bone	1 (2%)	0 (0%)	
clinoid	12 (18%)	5 (16%)	
clivus	1 (2%)	0 (0%)	
olfactory groove	8 (12%)	2 (7%)	
optic nerve sheath	3 (5%)	0 (0%)	
petroclival	0 (0%)	2 (7%)	
cavernous sinus	30 (45%)	4 (13%)	**0.006**
spheno-orbital	0 (0%)	4 (13%)	
tentorial	1 (2%)	0 (0%)	
tuberculum	5 (8%)	5 (16%)	
**Dose delivered to 1 mm^3^ optic nerve**			
Mean (SD)	17.3 (4.3)	18.3 (4.0)	0.189
Median [Min, Max]	17.5 [6.0, 27.0]	19.5 [7.5, 25.7]	
**Dose delivered to 1 mm^3^ optic chiasm**			
Mean (SD)	14.4 (61)	12.7 (6.5)	0.322
Median [Min, Max]	13.5 [3.8, 35.5]	14.0 [4.0, 24.0]	
**Visual disorders**			
no	45 (67%)	19 (61%)	0.623
yes	22 (33%)	12 (39%)	
**Visual field disorders**			
no	54 (81%)	23 (74%)	0.651
yes	13 (19%)	8 (26%)	
**Trigeminal dysesthesias**			
no	61 (91%)	28 (90%)	1.000
yes	6 (9%)	3 (10%)	
**III-IV-VI cn deficits**			
no	53 (79%)	28 (90%)	0.281
yes	14 (21%)	3 (10%)	
**Number of fraction**			
3	7 (10%)	2 (7%)	0.637
4	1 (2%)	0 (0%)	
5	59 (88%)	29 (93%)	
**Volume (cm^3^)**			
Mean (SD)	6.6 (5.3)	7.5 (8.1)	0.649
Median [Min, Max]	6.3 [0.1, 22.3]	4.2 [0.2, 31.7]	
**Clinical fu**			
Stable	61 (91%)	29 (94%)	0.847
Worsened	2 (3%)	1 (3%)	
Improved	4 (6%)	1 (3%)	
**Radiological fu**			
Increased	1 (2%)	0 (0%)	**0.035**
Reduced	19 (28%)	2 (7%)	
Stable	47 (70%)	29 (93)	

Bolded *p*-values indicate statistically significant results (*p* < 0.05).

## Data Availability

The raw/processed data required to reproduce the above findings cannot be shared at this time due to legal/ethical reasons.
